# Advances in research on the effectiveness and mechanism of Traditional Chinese Medicine formulas for colitis-associated colorectal cancer

**DOI:** 10.3389/fphar.2023.1120672

**Published:** 2023-02-23

**Authors:** Xiunan Wei, Xiaohui Leng, Gongyi Li, Ruting Wang, Lili Chi, Dajuan Sun

**Affiliations:** ^1^ College of First Clinical Medicine, Shandong University of Traditional Chinese Medicine, Jinan, China; ^2^ Weifang Traditional Chinese Hospital, Weifang, China; ^3^ College of Chinese Medicine, Shandong University of Traditional Chinese Medicine, Jinan, China; ^4^ Affiliated Hospital of Shandong University of Traditional Chinese Medicine, Jinan, China

**Keywords:** colitis-associated colorectal cancer, inflammatory bowel disease, traditional Chinese medicine formula, mechanism, review

## Abstract

Inflammatory bowel disease (IBD) can progress into colitis-associated colorectal cancer (CAC) through the inflammation-cancer sequence. Although the mechanism of carcinogenesis in IBD has not been fully elucidated, the existing research indicates that CAC may represent a fundamentally different pathogenesis pattern of colorectal cancer. At present, there is no proven safe and effective medication to prevent IBD cancer. In recent years, Chinese medicine extracts and Chinese medicine monomers have been the subject of numerous articles about the prevention and treatment of CAC, but their clinical application is still relatively limited. Traditional Chinese Medicine (TCM) formulas are widely applied in clinical practice. TCM formulas have demonstrated great potential in the prevention and treatment of CAC in recent years, although there is still a lack of review. Our work aimed to summarize the effects and potential mechanisms of TCM formulas for the prevention and treatment of CAC, point out the issues and limitations of the current research, and provide recommendations for the advancement of CAC research in the future. We discovered that TCM formulas regulated many malignant biological processes, such as inflammation-mediated oxidative stress, apoptosis, tumor microenvironment, and intestinal microecology imbalance in CAC, through a review of the articles published in databases such as *PubMed, SCOPUS, Web of Science, Embase, and CNKI*. Several major signal transduction pathways, including NF-κB, STAT3, Wnt/β-catenin, HIF-1α, and Nrf2, were engaged. TCM formula may be a promising treatment candidate to control the colitis-cancer transformation, however further high-quality research is required.

## 1 Introduction

Inflammatory bowel disease (IBD) is a chronic, lifelong, progressive, and difficult-to-cure disease (Kaplan, 2015). It primarily consists of Crohn’s disease (CD), ulcerative colitis (UC), and uncertain colitis. Over the past 30 years, the incidence and prevalence of IBD have increased in China and other Asian countries. According to a South Korea survey, the incidence of UC increased by approximately 20 times, and that of CD increased by about 40 times for 30 years from 1986 to 2015 (Aniwan et al., 2022). A Chinese survey revealed that the number of IBD cases in China increased by approximately 3 times from 1990 to 2019 (Shao et al., 2022). Notably, the incidence rate is predicted to continue in the next 25 years (Shao et al., 2022). The prevalence of IBD has increased globally, accompanied by a significant financial burden and cost of care (GBD 2017 Inflammatory Bowel Disease Collaborators, 2020). IBD can progress into colitis-associated colorectal cancer (CAC) through the sequence evolution process of “chronic inflammatory reaction-low grade dysplasia-high grade dysplasia-cancer” (Eaden et al., 2001; Rogler, 2014). As one of the dangerous complications of IBD, CAC accounts for approximately 15% of all deaths of patients with IBD (Munkholm, 2003). At the same time, CAC is also the primary cause of colectomy in IBD patients (Mattar et al., 2011). A recent epidemiological survey of 390,624 patients with colorectal cancer (CRC) revealed that CAC accounts for approximately 1.3% of CRC (Birch et al., 2022). In contrast with sporadic colorectal cancer, CAC has an earlier diagnosis age (Munkholm, 2003), a higher proportion of multiple cancers, and a worse prognosis (Watanabe et al., 2011).

Combined with previous reports, the activation of some classical pathways and abnormal expression of some cytokines promote carcinogenesis of IBD-CAC, including nuclear transcription factor-kappa B(NF-кB), Signal transducer and activator of transcription 3(STAT3), wingless/integrated (Wnt)/β-catenin, interleukin (IL)-6, IL-1β, and IL-17 (Hirano et al., 2020). However, drugs targeting specific pathways and cytokines remain unavailable, and there is no randomized controlled clinical trial on chemical prophylaxis of IBD-CAC. Retrospective observation studies of different qualities provide evidence for prophylaxis and treatment of CAC. Although preparations (van Staa et al., 2005) and immunomodulators (Zhu et al., 2018) containing 5-aminosalicylic acid (5-ASA) can partially regulate the progress of inflammation, their prophylaxis and treatment of CAC have controversial results (Bernstein et al., 2011; Jess et al., 2014). Although the use of biological agents has initiated a new era of IBD treatment, there exist still certain issues, including increased tumor risk (Cushing and Higgins, 2021), induction of infection (Cushing and Higgins, 2021), and drug non-responsiveness (Schnitzler et al., 2009). Therefore, we can only presently monitor carcinogenesis risk of IBD through regular endoscopic examination and pathological biopsy; besides, effective drugs to prevent IBD from converting to CAC are still lacking.

Traditional Chinese medicine (TCM) has demonstrated significant potential in prophylaxis and treatment of CAC in the past few years. TCM formulas can reflect the benefits of TCM in the treatment of multiple components, channels, targets, and synergy as well as attenuation between drugs, however, the mechanisms of action are often unclarified, significantly limiting clinical promotion. In the past 5 years, studies on the mechanisms of TCM formula prophylaxis and treatment of CAC have been emerging, however, relevant reviews are lacking. We set the keyword as the combination of *“colitis-associated cancer”* and *“Chinese medicine”* and searched databases including PubMed, SCOPUS, Web of Science, Embase, and CNKI to clarify the efficacy of TCM formulas and molecular mechanisms of their effects. This paper will first introduce the peculiar pathogenesis of CAC, different from sporadic CRC, before reviewing the research progress in prophylaxis and treatment of CAC with TCM formulas.

## 2 Pathogenesis process of CAC

Although the mechanism of carcinogenesis in IBD has not been fully elucidated, research has identified that persistent histological inflammation is one of the conditions mediating the development of CAC (Jess et al., 2012; Yvellez et al., 2021). One meta-analysis has shown that the probability of CRC increases with the prolongation of the disease process in UC patients. Cancer rates in the 10-year, 20-year and 30-year periods following the onset of UC include 1.6%, 8.3% and 18.4%, respectively (Eaden et al., 2001). Another retrospective study has also shown that the cumulative inflammatory burden (CIB), including the duration and severity of IBD, increases the risk of CRC (Choi et al., 2019). The risk of cancer increases two times in 10-year-old, 5-year-old moderate, and 3.3-year-old severe persistent active colitis (Choi et al., 2019). A recent large multi-cohort retrospective study from the United Kingdom confirmed that patients with large, uncut and multifocal low-grade intraepithelial neoplasia and recent moderate or severe active inflammation of UC had the highest risk of developing advanced neoplasia (high-grade dysplasia or CRC) (Curtius et al., 2022). In IBD cases, a larger area of inflamed mucosa is more likely to transform into a tumor in the process of “field cancerization” (Rubin et al., 1992). Accumulating studies have shown that chronic inflammation continuously stimulates colonic epithelial cells and induces dysplasia *via* various mechanisms, including induction of genetic changes (Baker et al., 2018), oxidative stress-driven DNA damage (Fantini and Guadagni, 2021), abnormal immune responses, and destruction of the intestinal flora (Grivennikov, 2013), strongly promoting the occurrence and development of CAC.

CRC is caused by the loss of the tumor suppressor gene APC, resulting in permanent activation of the Wnt pathway (van Neerven et al., 2021). As a clinical subtype of CRC, CAC has numerous similar molecular mechanisms to CRC, including: 1) both of them originate from abnormally developed precursor lesions; 2) the mutation frequencies of key genes including adenomatous polyposis coli (APC), tumor suppressor protein p53 (p53), small mothers against decapentaplegic 4 (SMAD4), and kirsten rat sarcoma virus (KRAS) causing carcinogenesis between the two are equivalent (Clevers, 2004). However, unlike sporadic CRC, CAC exhibits three specific morphological and molecular features. First, in contrast with precancerous lesions, adenomas with protruding features of sporadic CRC-precursor lesions of CAC often have flat morphological features (Porter et al., 2021). Secondly, sporadic adenoma dysplastic cells often occupy the upper part of the tumor recess, whereas dysplastic cells in colitis usually involve the entire crypt (Shah and Itzkowitz, 2022). Thirdly, mutations and deletions of p53, SMAD4, and deleted in colon cancer (DCC) occur early in CAC, whereas the loss of APC and KRAS mutations occur late in CAC, exactly the opposite of the period in which sporadic CRC molecular mutations occur (Nagao-Kitamoto et al., 2022). A transcriptome-based study of tumor subtypes revealed that the classical Wnt signaling-related epithelial tumor subtype (CMS2) was completely absent in IBD-associated CRC and was dominated by the mesenchymal subtype (CMS4) (Rajamäki et al., 2021). This suggests that epithelial-mesenchymal transition (EMT) may occupy an important position in the carcinogenesis of IBD, and CAC may represent a unique tumorigenesis pathway, affecting the prognosis and therapy plan for CAC patients treated with drugs against CRC.

## 3 The roles of TCM formulas in the prophylaxis and treatment of CAC

**FIGURE 1:**
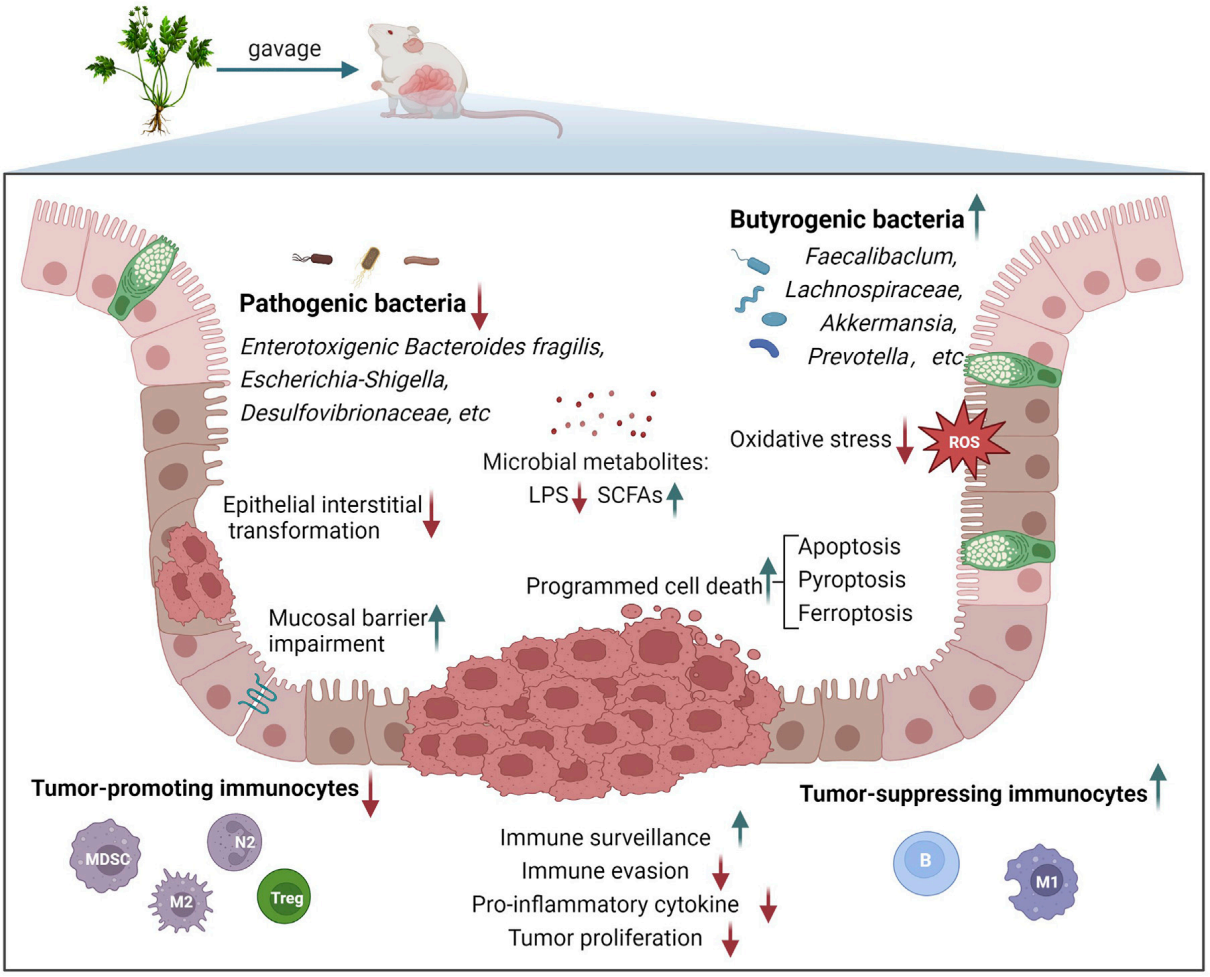
Main biological processes involved in the treatment of CAC with TCM formulas.

Research on intervention effects and mechanisms of TCM formulas on CAC has been primarily confirmed in basic experiments. AOM/DSS-induced CAC is currently the most commonly adopted *in vivo* model for studying the drug prophylaxis and treatment mechanism of CAC. Through intraperitoneal injection of the carcinogenic agent azoxymethane (AOM) and free drinking of 3 cycles of dextran sodium sulfate (DSS) by experimental rats, this model properly simulated the conversion process of IBD to CAC in the environment of chronic inflammation (Clevers, 2004). At present, this method is quite mature and has been broadly applied in efficacy evaluation and mechanistic research for prophylaxis and treatment of CAC (Greten et al., 2004; Malik et al., 2018; Guo et al., 2021). In the CAC animal model, TCM formulas influence various abnormal processes in the incidence and progression of CAC by regulating the inflammatory process, also reflecting multichannel and multitarget features. In this paper, we found eighteen TCM formulas for the prevention and treatment of CAC (Table 1 **)**. Generally, TCM formulas for preventing and treating CAC majorly regulate the biological processes, including inflammation-mediated oxidative stress, apoptosis, the tumor microenvironment (TME), and intestinal microecology imbalance (Figure 1).

**TABLE 1:** Lists of TCM formulas with potential anti-CAC action.

Overall mechanism	No.	TCM formula	Composition	Extraction	Optimal dose	Model	Positive control	Administration route	Effects and potential mechanism	Ref
Inhibition of Oxidative stress	1	ShaoYao decoction (SYD)	9: *Paeonia lactiflora Pall.* [Paeoniaceae; Paeoniae radix alba], *Angelica sinensis* (Oliv.) Diels [Apiaceae; Angelicae sinensis radix], *Coptis chinensis Franch.* [Ranunculaceae; Coptidis rhizoma], *Areca catechu L.* [Arecaceae; Arecae semen], *Dolomiaea costus* (Falc.) Kasana and A.K.Pandey [Asteraceae; Aucklandiae radix], *Glycyrrhiza uralensis* Fisch. ex DC. [Fabaceae; Glycyrrhizae radix et rhizoma], *Rheum officinale Baill.* [Polygonaceae; Rhei radix et rhizoma], *Scutellaria baicalensis Georgi* [Lamiaceae; Scutellariae radix], *Cinnamomum tamala* (Buch.-Ham.) T.Nees and C.H.Eberm. [Lauraceae; Cinnamomi cortex]	Mix in a ratio of 30: 15: 15: 6: 6: 6: 9: 25: 7.5 g in sequence	37 g/kg	AOM/DSS induced CAC	SASP	oral gavage	alleviating colorectal carcinoma tumor progression, anti-inflammatory, resisting oxidative stress by activating Keap1-Nrf2-ARE signal	Wang et al. (2019)
				8 times the volume (v/m) of distilled water was added and stirred at 80°C for 1 h to obtain an aqueous extract. The supernatant was collected by centrifugation and concentrated at 70°C						
	2	Wumei Pill (WMP)	10: *Prunus mume* (Siebold) Siebold and Zucc. [Rosaceae; Mume fructus], *Asarum heterotropoides* F.Schmidt [Aristolochiaceae; Asari radix et rhizoma], *Zingiber officinale Roscoe* [Zingiberaceae; Zingiberis rhizoma], *Coptis chinensis Franch.* [Ranunculaceae; Coptidis rhizoma], *Angelica sinensis* (Oliv.) Diels [Apiaceae; Angelicae sinensis radix], *Aconitum carmichaelii Debeaux* [Ranunculaceae; Aconiti lateralis radix praeparata], *Zanthoxylum bungeanum Maxim.* [Rutaceae; Zanthoxyli pericarpium], *Neolitsea cassia* (L.) Kosterm. [Lauraceae; Cinnamomi ramulus], Panax ginseng C.A.Mey. [Araliaceae; Ginseng radix et rhizoma], *Phellodendron amurense Rupr.* [Rutaceae; Phellodendri chinensis cortex]	Drug proportion and extraction process not provided	6 g/kg	AOM/DSS induced CAC	Oxaliplatin	oral gavage	ameliorating CAC, inhibiting inflammatory and oxidative stress reactions, inhibiting AHCY expression by suppressing Hedgehog signal	Wang et al. (2022a)
	3	Huangqin decoction (HQD)	4: *Scutellaria baicalensis Georgi* [Lamiaceae; Scutellariae radix], *Paeonia lactiflora Pall.* [Paeoniaceae; Paeoniae radix alba], *Glycyrrhiza uralensis* Fisch. ex DC. [Fabaceae; Glycyrrhizae radix et rhizoma], *Ziziphus jujuba* Mill. [Rhamnaceae; Jujubae fructus]	Mix in a ratio of 9: 6: 6: 49 g in sequence	9.1 g/kg	AOM/DSS induced CAC	none	oral gavage	ameliorating chronic UC and preventing CAC, suppressing inflammatory cytokine production, and improving antioxidative activity	Chen et al. (2016)
				HQD-1: 10 times volume and 8 times volume of distilled water were added, respectively, at 100°C for 30min. Then that two liquids were combined						
				HQD-2: Four different herbal extracts were extracted under similar conditions, and then four herbal extracts were combined						
				HQD-3 and HQD-4: Prepared in 70% ethanol at 60°C similar to HQD-1 and HQD-2, respectively						
Induction of epithelial apoptosis	4	Huangqi Baizhu decoction (HBD)	2: *Astragalus mongholicus Bunge* [Fabaceae; Astragali radix], *Atractylodes lancea* (Thunb.) DC. [Asteraceae; Atractylodis rhizoma]	Mix in a ratio of 3: 1 in sequence	12 g/kg	AOM/DSS induced CAC	SASP	oral gavage	inhibiting occurrence and tumor proliferation of CAC, attenuating expression of inflammatory factors, and IL-6/STAT3/Survivin/CyclinD1 signal	Deng et al. (2019a)
				Soaked in 10 times volume of pure water for 30min. The water was removed and filter. The mixture was concentrated to 100 mL in a vacuum spin evaporator and dried in a freeze dryer to a frozen powder						
			*Atractylenolide II, Astragaloside*		50 μg/mL	IL-6 induced RAW264.7 and HCT-116 cells	None	added in cell culture medium	suppressing STAT3 activation by inhibiting activity of IL-6Rα/STAT3 signal, promoting apoptosis	
	5	Chang Qing formula (CQF)	10: *Cuscuta australis* R.Br. [Convolvulaceae; Cuscutae semen], *Epimedium brevicornu Maxim.* [Berberidaceae; Epimedii folium], *Angelica sinensis* (Oliv.) Diels [Apiaceae; Swertiae herba], *Pseudostellaria heterophylla* (Miq.) Pax [Caryophyllaceae; Pseudostell ariae radix], *Taraxacum mongolicum Hand.-Mazz.* [Asteraceae; Typhae pollen], *Phellodendron chinense C.K.Schneid.* [Rutaceae; Phellodendri chinensis cortex], *Bupleurum scorzonerifolium Willd.* [Apiaceae; Bupleuri radix], *Pinellia ternata* (Thunb.) Makino [Araceae; Pinelliae rhizoma], *Prunus armeniaca var. armeniaca* [Rosaceae; Armeniacae semen amarum], *Glycyrrhiza uralensis* Fisch. ex DC. [Fabaceae; Glycyrrhizae radix et rhizoma]	Mix in a ratio of 30: 30: 30: 30: 21: 12: 12: 12: 12: 3 g in sequence	6.25 g/kg	AOM/DSS induced CAC	none	oral gavage	reducing tumor numbers and tumor size, reducing IL-17A levels, inhibiting NF-κB/IL-6/STAT3 signaling cascade, suppressing MMP9 expression, and promoting tumor cell apoptosis	Luo et al. (2022)
				2300 mL pure water was added, recovered after heating for 1h, and then filtered. This process was repeated 2 times to obtain a mixed decoction. Finally, the mixture was concentrated and dried by a rotary evaporator to obtain 30.4 g of the extract						
	6	Feng-Liao-Chang-Wei-Kang (FLCWK)	*2: *Daphniphyllum calycinum Benth.* [Daphniphyllaceae], *Persicaria hydropiper* (L.) Delarbre [Polygonaceae]*	Drug proportion and extraction process not provided	9 g/kg	AOM/DSS induced CAC	5-FU	oral gavage	reducing colon tumors, increasing survival rate, preventing IL-6/STAT3 signal	Zhong et al. (2020)
					80 μg/mL	IL-6 induced HCT116 cells	5-FU	added in cell culture medium	the inhibiting proliferation of tumor cells, promoting apoptosis by blocking the IL-6/STAT3 signal	
	7	Compound sophorae decoction (CSD)	6: *Sophora flavescens Aiton* [Fabaceae; Sophorae flavescentis radix], *Sanguisorba officinalis L.* [Rosaceae; Sanguisorbae radix], *Strobilanthes cusia* (Nees) Kuntze [Acanthaceae; Indigo naturalis], *Bletilla striata* (Thunb.) Rchb.f. [Orchidaceae; Bletillae rhizoma], *Panax notoginseng* (Burkill) F.H.Chen [Araliaceae; Notoginseng radix et rhizoma], *Glycyrrhiza uralensis Fisch.* ex DC. [Fabaceae; Glycyrrhizae radix et rhizoma]	Mix in a ratio of 15∶15∶3∶10∶3∶10 g in sequence	0.1614 g/d	AOM/DSS induced CAC	none	oral gavage	allaying malignancy, moderating malignant inflammatory features, modulating apoptosis and mitophagy	Deng et al. (2019b)
				Boiled for 1 h and concentrated the mixture to a CSD concentration of 1.076 g/ml						
	8	Yipi Yangwei decoction (YYD)	10: *Codonopsis pilosula* (Franch.) Nannf. [Campanulaceae; Codonopsis radix], *Prunus mume* (Siebold) Siebold and Zucc. [Rosaceae; Linderae radix], *Atractylodes lancea* (Thunb.) DC. [Asteraceae; Atractylodis rhizoma], *Curcuma phaeocaulis Valeton* [Zingiberaceae; Curcumae rhizoma], *Dioscorea oppositifolia L.* [Dioscoreaceae; Dioscoreae rhizoma], *Poria cocos* (Schw.) Wolf [Polyporaceae; Poria], *Coix lacryma-jobi* var. ma-yuen (Rom.Caill.) Stapf [Poaceae; Coicis semen], *Citrus × aurantium* f. deliciosa (Ten.) M.Hiroe [Rutaceae; Citri reticulatae pericarpium], *Magnolia officinalis* Rehder and E.H.Wilson [Magnoliaceae; Magnoliae officinalis cortex], *Glycyrrhiza uralensis* Fisch. ex DC. [Fabaceae; Glycyrrhizae radix et rhizoma]	Mix in a ratio of 10:6:10:6:15:15:30:6:9:3 g in sequence	17.16 g/kg	AOM/DSS induced CAC	none	enema	preventing CAC, inhibiting TLR4/MAPKS/NF-κB signal, downregulating PCNA, COX-2, and Bcl-xL, regulating inflammatory cytokines	Lou et al. (2018)
				Appropriate amount of distilled water was added to obtain Chinese medicinal liquid, and concentrated to 1 g/mL						
	9	Huangqin Tang (HQT)	4: *Scutellaria baicalensis Georgi* [Lamiaceae; Scutellariae radix], *Paeonia lactiflora Pall.* [Paeoniaceae; Paeoniae radix alba], *Glycyrrhiza uralensis* Fisch. ex DC. [Fabaceae; Glycyrrhizae radix et rhizoma], *Ziziphus jujuba Mill.* [Rhamnaceae; Jujubae fructus]	Mix in a ratio of 3:2:2:3 in sequence	20 g/kg	AOM/DSS induced CAC	none	oral gavage	ameliorating CAC symptoms, decreasing inflammatory cytokines, regulating EMT and cell cycle	Ma et al. (2022)
				Boiled with ddH2O at 100°Ctwice (1:10, w/v) (1:8, w/v), each time for 1 h. Collect and combine the filtrates of the two groups. Then the filtrate was completely dried under reduced pressure to obtain an extract with a concentration of 1 g/ml						
Regulation of tumor microenvironment	10	Huang Qin decoction (HQD)	4: *Scutellaria baicalensis Georgi* [Lamiaceae; Scutellariae radix], *Paeonia lactiflora Pall.* [Paeoniaceae; Paeoniae radix alba], *Glycyrrhiza uralensis* Fisch. ex DC. [Fabaceae; Glycyrrhizae radix et rhizoma], *Ziziphus jujuba Mill.* [Rhamnaceae; Jujubae fructus]	Drug proportion not provided	1.5 g/kg	AOM/DSS induced CAC	Aspirin	oral gavage	restricting the formation of intestinal tumors, reducing neutrophil infiltration, enhancing immune surveillance of CD8^+^ T-cell by targeting PAD4-dependent NETs	Pan et al. (2022)
				Soaked in 8 times water for 20 min, boiled for 25 min and simmered for 35 min. The 2 part of that filtrate were mixed and evaporate cyclically to a concentration of 1 g/ml						
	11	Xian-Lian-Jie-Du decoction (XLJDD)	4: *Agrimonia pilosa Ledeb.* [Rosaceae; Agrimoniae herba], *Coptis chinensis Franch.* [Ranunculaceae; Coptidis rhizoma], *Astragalus mongholicus Bunge* [Fabaceae; Astragali radix], *Coix lacryma-jobi* var. ma-yuen (Rom.Caill.) Stapf [Poaceae; Coicis semen]	Drug proportion not provided	12.9 g/kg	AOM/DSS and high-fat diet-induced CAC	Folic Acid	oral gavage	preventing CAC by activating the expression of Mfsd2a and Ccdc85c, reducing infiltration of B-cell in CAC microenvironment	Yu et al. (2022)
				Immersed in water (1:10, w/v) for 0.5 h and boiled for 2 h. The aqueous extract was filtered and the residue was decocted again with water (1: 8, w/v) under the same conditions. The filtrate was combined and precipitate with 60% alcohol for 1 day. Concentrate to 2 g/ml in a rotary evaporator						
	12	Yi-Yi-Fu-Zi-Bai-Jiang-San (YYFZBJS)	3: *Coix lacryma-jobi* var. ma-yuen (Rom.Caill.) Stapf [Poaceae; Coicis semen], *Aconitum carmichaelii Debeaux* [Ranunculaceae; Aconiti lateralis radix praeparata], *Patrinia scabiosifolia* Link [Caprifoliaceae]	Drug proportion not provided	15.3 g/kg	AOM/DSS induced CAC	Aspirin	oral gavage	suppressing colorectal carcinogenesis by restoring immune suppressive activity, suppressing HIF-1α expression, and improving immune responses	Zhang et al. (2022)
				Added appropriate water, extracted 2 times, filtered, and dried to obtain dried extract						
					62.5 μg/ml	HIF-1α induced Tregs coincubated with MC38, IEC-6	none	added in cell culture medium	inhibiting CRC cell proliferation through regulating HIF-1α transfected regulatory T-cell	
	13	Shenling Baizhu San (SBS)	10: *Panax ginseng* C.A.Mey. [Araliaceae; Ginseng radix et rhizoma], *Poria cocos* (Schw.) Wolf [Polyporaceae; Poria], *Atractylodes macrocephala Koidz.* [Asteraceae; Atractylodis macrocephalae rhizoma], *Lablab purpureus* subsp. purpureus [Fabaceae; Lablab semen album], *Dioscorea oppositifolia L.* [Dioscoreaceae; Dioscoreae rhizoma], *Glycyrrhiza uralensis* Fisch. ex DC. [Fabaceae; Glycyrrhizae radix et rhizoma], *Nelumbo nucifera Gaertn.* [Nelumbonaceae; Nelumbinis semen], *Wurfbainia villosa* (Lour.) Škorničk. and A.D.Poulsen [Zingiberaceae; Amomi fructus], *Coix lacryma-jobi* var. ma-yuen (Rom.Caill.) Stapf [Poaceae; Coicis semen], *Platycodon grandiflorus* (Jacq.) A.DC. [Campanulaceae; Platycodonis radix]	Mix in a ratio of 15: 15: 15: 12: 15: 9: 9: 6: 9: 6 in sequence	7.28 g/kg	AOM/DSS induced CAC	none	oral gavage	reducing colonic neoplasms, decreasing MDSCs infiltration and TGF-β1, ameliorating immunosuppressive tumor microenvironment	Lin et al. (2015)
				10 times of distilled water (v/m) was added and stirred at 80°C for 1 h for extraction. Then centrifuged at 1,500×g at room temperature, collected supernatant and concentrated under reduced pressure at 70°C						
					16 mg/mL	TGF-β1 induced CRC cells	Ginsenoside Rc, Atractylenolide-1	added in cell culture medium	reducing CRC cell viability, downregulating the Wnt signaling pathway, inhibiting EMT	
	14	Shaoyao decoction (SYD)	9: *Paeonia lactiflora Pall.* [Paeoniaceae; Paeoniae radix alba], *Angelica sinensis* (Oliv.) Diels [Apiaceae; Angelicae sinensis radix], *Coptis chinensis Franch.* [Ranunculaceae; Coptidis rhizoma], *Areca catechu L.* [Arecaceae; Arecae semen], *Dolomiaea costus* (Falc.) Kasana and A.K.Pandey [Asteraceae; Aucklandiae radix], *Glycyrrhiza uralensis* Fisch. ex DC. [Fabaceae; Glycyrrhizae radix et rhizoma], *Rheum officinale Baill.* [Polygonaceae; Rhei radix et rhizoma], *Scutellaria baicalensis Georgi* [Lamiaceae; Scutellariae radix], *Cinnamomum tamala* (Buch.-Ham.) T.Nees and C.H.Eberm. [Lauraceae; Cinnamomi cortex]	Mix in a ratio of 30: 15: 15: 6: 6: 6: 9: 15: 7.5 in sequence	7.12 g/kg	AOM/DSS induced CAC	none	oral gavage	reducing incidence and multiplicity of CAC, repressing inflammatory cytokines, inhibiting Snail-induced EMT by attenuating TAM infiltration and NF-κB activation	Lin et al. (2014)
				10 times of distilled water (v/m) was added and stirred at 80°C for 1 h for extraction. Then centrifuged at 1,500×g at room temperature, collected supernatant and concentrated under reduced pressure at 70°C						
					8 mg/mL	CRC cells	Paeonol	added in cell culture medium	inhibiting CRC cell proliferation, decreasing oncogenic proteins	
Regulation of the intestinal microbiota and its metabolites	15	Huoxiang Zhengqi (HXZQ)	10: *Atractylodes macrocephala Koidz.* [Asteraceae; Atractylodis macrocephalae rhizoma], *Citrus × aurantium* f. deliciosa (Ten.) M.Hiroe [Rutaceae; Citri reticulatae pericarpium], *Magnolia officinalis* Rehder and E.H.Wilson [Magnoliaceae; Magnoliae officinalis cortex], *Angelica dahurica* (Hoffm.) Benth. and Hook.f. ex Franch. and Sav. [Apiaceae; Angelicae dahuricae radix], Poria cocos (Schw.) Wolf [Polyporaceae; Poria], *Areca catechu L.* [Arecaceae; Arecae pericarpium], *Pinellia ternata* (Thunb.) Makino [Araceae; Pinelliae rhizoma], *Glycyrrhiza uralensis* Fisch. ex DC. [Fabaceae; Glycyrrhizae radix et rhizoma], Oleum Pogostemonis, Oleum Folii Perillae	Mix in a ratio of 160 g: 160 g: 160 g: 240 g: 240 g: 240 g: 160 g: 20 g: 1.6 ml: 0.8 ml in sequence	1.35 g/kg	AOM/DSS induced CAC	none	oral gavage	inhibiting tumor growth, regulating intestinal microbiota, altering metabolism, suppressing inflammatory and oxidative response through Nrf2/NF-κB signaling	Dong et al. (2022)
				Extraction process not provided						
	16	Chang-wei-qing (CWQ)	8: *Astragalus mongholicus Bunge* [Fabaceae; Astragali radix], *Atractylodes lancea* (Thunb.) DC. [Asteraceae; Atractylodis rhizoma], *Codonopsis pilosula* (Franch.) Nannf. [Campanulaceae; Codonopsis radix], *Akebia quinata* (Thunb. ex Houtt.) Decne. [Lardizabalaceae; Akebiae Caulis], *Polyporus umbellatus* (Pers.) Fries [Polyporaceae; Polyporus], *Coix lacryma-jobi* var. ma-yuen (Rom.Caill.) Stapf [Poaceae; Coicis semen], *Causonis japonica* (Thunb.) Raf. [Vitaceae], *Sargentodoxa cuneata* (Oliv.) Rehder and E.H.Wilson [Lardizabalaceae; Sargentodoxae caulis]	Mix in a ratio of 10: 5: 5: 8: 8: 10: 10 in sequence	10 mg/kg	AOM/DSS induced CAC	Bifico	oral gavage	ameliorating tumor development, mitigating pro-inflammatory response, decreasing TLR4/NF-κB and STAT3 signaling pathway, influencing microbiota composition, improving integrity of intestinal mucosa	Wan et al. (2019)
				Decocted with 3000 mL water twice, 1 h each time, and filtered						
	17	San-Wu-Huang-Qin decoction (SWHQ)	*Astragalus mongholicus Bunge* [Fabaceae; Astragali radix], *Atractylodes lancea* (Thunb.) DC. [Asteraceae; Atractylodis rhizoma], *Codonopsis pilosula* (Franch.) Nannf. [Campanulaceae; Codonopsis radix], *Akebia quinata* (Thunb. ex Houtt.) Decne. [Lardizabalaceae; Akebiae Caulis], *Polyporus umbellatus* (Pers.) Fries [Polyporaceae; Polyporus], *Coix lacryma-jobi* var. ma-yuen (Rom.Caill.) Stapf [Poaceae; Coicis semen], *Causonis japonica* (Thunb.) Raf. [Vitaceae], *Sargentodoxa cuneata* (Oliv.) Rehder and E.H.Wilson [Lardizabalaceae; Sargentodoxae caulis]	Mix in a ratio of 1: 1: 2 in sequence	7.28 g/kg	AOM/DSS induced CAC	Aspirin	oral gavage	inhibiting tumorigenesis, restoring colonic barrier integrity, reversing gut dysbiosis, suppressing TLR4/NF-kB signaling pathway	Zhou et al. (2022)
				10 times of distilled water (W/V) was added, and the mixture was immersed at room temperature for 1 h. Then boiled and maintained at 100°C for 1 h, and the liquid medicine was filtered and collected. Then 8 times of distilled water (W/V) was added and the mixture was decocted for 45 min. The two liquids were mixed again and concentrated in a rotary evaporator at 60°C						
	18	Wu Mei Wan (WMW)	10: *Prunus mume* (Siebold) Siebold and Zucc. [Rosaceae; Mume fructus], *Asarum heterotropoides* F.Schmidt [Aristolochiaceae; Asari radix et rhizoma], *Zingiber officinale Roscoe* [Zingiberaceae; Zingiberis rhizoma], *Coptis chinensis Franch.* [Ranunculaceae; Coptidis rhizoma], *Angelica sinensis* (Oliv.) Diels [Apiaceae; Angelicae sinensis radix], *Aconitum carmichaelii Debeaux* [Ranunculaceae; Aconiti lateralis radix praeparata], *Zanthoxylum bungeanum Maxim.* [Rutaceae; Zanthoxyli pericarpium], *Neolitsea cassia* (L.) Kosterm. [Lauraceae; Cinnamomi ramulus], *Panax ginseng* C.A.Mey. [Araliaceae; Ginseng radix et rhizoma], *Phellodendron amurense Rupr.* [Rutaceae; Phellodendri chinensis cortex]	Mix in a ratio of 8: 8: 3: 3: 3: 3: 2: 5: 3: 2 in sequence	5.8 g/kg	AOM/DSS induced CAC	none	oral gavage	attenuating CAC by regulating the balance between “tumor-promoting bacteria” and “tumor-suppressing bacteria” and NF-kB/IL-6/STAT3 pathway	Jiang et al. (2020)
				Boiled in 1800 ml distilled water, refluxed, extracted and concentrated to 0.5 g/ml						
	19	Canmei formula (CMF)	2: *Prunus mume* (Siebold) Siebold and Zucc. [Rosaceae; Mume fructus], *Bombyx mori Linnaeus* [Bombycidae; Bombyx batryticatus]	Mix in a ratio of 1:1 in sequence	0.657 g/kg (Ethanol extract)	AOM/DSS and high-fat diet-induced CAC	Aspirin	oral gavage	attenuating AOM/DSS-induced colitis-associated tumorigenesis, repressing NF-κB and IL-17C signaling, regulating gut microbiota	Zhang et al. (2019b)
				Aqueous extract: 980 g of the mixture was extracted twice for 1.5 h and 1 h. The filtrate was concentrated and dried under vacuum at 60°C						
				Alcohol extract: the same procedure was repeated twice in the CMF extract. Then washed with pure water, 20% ethanol and 90% ethanol, precipitated, concentrated and dried in a vacuum at 60°C						
	20	Qingchang Wenzhong decoction (QCWZD)	8: *Coptis chinensis Franch.* [Ranunculaceae; Coptidis rhizoma], *Zingiber officinale Roscoe* [Zingiberaceae; Zingiberis rhizoma praeparatum], *Sophora flavescens Aiton* [Fabaceae; Sophorae flavescentis radix], *Dolomiaea costus* (Falc.) Kasana and A.K.Pandey [Asteraceae; Aucklandiae radix], *Basella alba L.* [Basellaceae; Notoginseng radix et rhizoma], *Strobilanthes cusia* (Nees) Kuntze [Acanthaceae; Indigo naturalis], *Sanguisorba officinalis L.* [Rosaceae; Sanguisorbae radix], *Glycyrrhiza uralensis* Fisch. ex DC. [Fabaceae; Glycyrrhizae radix et rhizoma]	Drug proportion and extraction process not provided	1.4 g/kg	(AOM/DSS)- and Apc^min/+^ induced tumor	none	oral gavage	preventing the occurrence of intestinal tumors by improving the function of the intestinal barrier and inhibiting GSDME-mediated pyroptosis	Ren et al. (2022)
	21	Pai-Nong-San (PNS)	3: *Citrus × aurantium L.* [Rutaceae; Aurantii fructus immaturus], *Paeonia lactiflora Pall.* [Paeoniaceae; Paeoniae radix alba], *Platycodon grandiflorus* (Jacq.) A.DC. [Campanulaceae; Platycodonis radix]	Mix in a ratio of 5:5:2 in sequence	3.2 g/kg	AOM/DSS induced CAC	Aspirin	oral gavage	inhibiting Wnt signaling, regulating disturbance of gut microbiota, reducing the number of CD4^+^ and CD8^+^ T lymphocytes, decreasing expression of IL-6, TNF-α, and HIF-α	Zhang et al. (2021b)
				The medicinal materials were pulverized, sieved, and suspended in pure water before administration						
	22	Yi-Yi-Fu-Zi-Bai-Jiang-San (YYFZBJS)	3: *Coix lacryma-jobi* var. ma-yuen (Rom.Caill.) Stapf [Poaceae; Coicis semen], *Aconitum carmichaelii Debeaux* [Ranunculaceae; Aconiti lateralis radix praeparata], *Patrinia scabiosifolia* Link [Caprifoliaceae]	Mix in a ratio of 30:6:15 in sequence	15.3 g/kg	AOM/DSS induced CAC	Aspirin	oral gavage	suppressing colorectal tumorigenesis and modulating gut microbiome composition, inhibiting ETBF-induced colorectal tumor development	Chai et al. (2021)
				The mixture (51 g) was extracted twice, 1 h each time, in ethanol (1:8, v/v) under reflux. The filtrate was concentrated and dried under vacuum at 60°C. The concentrate extract is then dried by freeze-dry						
						MC-38 cells	co-incubated with BMDMs of ETBF mice	co-incubated with BMDMs of ETBF + YYFZBJS mice	downregulating colony formation, decreasing c-Met, MMPs, and cyclinD1 expression, suppressing infiltration ability of tumor cells, decreasing phosphorylation of STAT3	
	23	Sini decoction (SND)	3: *Aconitum carmichaelii Debeaux* [Ranunculaceae; Aconiti lateralis radix praeparata], *Glycyrrhiza uralensis* Fisch. ex DC. [Fabaceae; Glycyrrhizae radix et rhizoma], *Zingiber officinale Roscoe* [Zingiberaceae; Zingiberis rhizoma]	Mix in a ratio of 103:385:512 g in sequence	3.5 g/kg	AOM/DSS induced CAC	none	oral gavage	intervening in CAC development by regulating immunity, protecting mucosal barrier, changing intestinal microbiota composition, decreasing inflammatory cytokines	Wang et al. (2021b)
				Boiled in 2L water for 60 min, then cool and boil for 40 min. Then the filtrate residue was discarded, and the filtrate was freeze-dried to make 300 g dry powder						

### 3.1 Inhibition of oxidative stress

Although little is known about the pathogenesis of IBD-CAC, inflammation and its resultant oxidative stress initiate IBD carcinogenesis. IBD is characterized by insufficient antioxidant defense capabilities and is considered a type of “oxy-overload” disease (Hofseth et al., 2003). The recurrence of this disease induces numerous infiltrated immune cells and the liberation of proinflammatory cytokines, causing colossal accumulation of reactive oxygen species (ROS) and reactive nitrogen species (RNS). ROS and RNS are produced by innate immune cells and induce oxidative stress reactions, resulting in cell DNA damage and increasing the permeability of the intestinal mucosal barrier to promote bacterial translocation (Lambert, 2009). Thus, this provides a basis for numerous carcinogenic events, including gene mutation (Wang et al., 2016), microsatellite instability (Hofseth et al., 2003), and abnormal methylation (Scarpa et al., 2014).

**Shaoyao decoction (SYD)**, originating from *Su-wen-bing-ji-qi-yi-bao-ming-ji*, is a classical prescription for the treatment of dysentery and comprises 9 TCM herbs, including *Paeonia lactiflora Pall.* [Paeoniaceae; Paeoniae radix alba] and *Angelica sinensis* (Oliv.) Diels [Apiaceae; Angelicae sinensis radix]. Wang et al. (Wang et al., 2019) intervened AOM/DSS induced mice with different doses of SYD aqueous extract (9.25, 18.5, and 37 g/kg) for 70 days. Sulfasalazine (SASP, 10 mg/kg) was used as the positive control. They discovered that SYD significantly suppressed weight loss and colonic shortening as well as reduced the number of tumors. The lowest effective dose of SYD was 9.25 g/kg and was dose-dependent. SYD could reduce serum tumor necrosis factor (TNF)-α, IL-6, IL-1β, and the expression of colonic Ki-67 as well as NF-κB, improving antioxidant stress capacity in the early stage. Nuclear factor erythroid 2-related factor 2 (Nrf2) is a key signaling molecule for oxidative stress (Piotrowska et al., 2021). Additionally, SYD activated Nrf2/ARE, and the 37 g/kg group was superior to the SASP group. This indicates that SYD can prevent and treat CAC and enhance the ability of anti-oxidative stress. As no targeted verification experiments have been designed in this study, it is still unclear whether SYD plays an anti-tumor role by resisting oxidative stress or regulating Nrf2/ARE.

**Wumei Pill (WMP)**, originating from *Shang-han-lun*, comprises 10 TCM herbs, including *Prunus mume* (Siebold) Siebold and Zucc. [Rosaceae; Mume fructus] and *Asarum heterotropoides* F. Schmidt [Aristolochiaceae; Asari radix et rhizoma]. It is used to treat chronic dysentery. Wang et al. (2022a) intervened AOM/DSS induced mice with different doses of WMP sodium carboxymethylcellulose (CMC-Na) resuspension (3, 6 g/kg) for 4 weeks. Oxaliplatin (5 mg/kg) was used as the positive control. The results showed that WMP at 6 g/kg could effectively suppress tumor proliferation, reduce the levels of IL-1β and IL-6, regulate oxidative stress markers malondialdehyde (MDA) and glutathione (GSH), as well as restore the activities of the antioxidant enzymes superoxide dismutase (SOD), catalase (CAT), and glutathione peroxidase (GSH-PX), and was superior to Oxaliplatin group. They also found abnormally high levels of S-adenosylhomocysteine hydrolase (AHCY) in human colon cancer and CAC mouse colon samples, whereas WMP suppressed AHCY and activation of Hedgehog (Hh)/Gli signaling cascade. The latest research indicates that Hh signaling pathway activates the pathogenesis of CAC (Zeng et al., 2022). In CRC cells cultured *in vitro*, AHCY overexpression activates the Hh signal, whereas its knockdown inactivates the Hh signal, thereby confirming the targeting relationship between the two. Further *in vitro* studies indicate that AHCY overexpression improves the carcinogenic functions of CRC cells, including proliferation, invasion, and angiogenesis, whereas its knock-out has the opposite effect, weakening cellular inflammation and oxidative stress. Of interest, subsequent experiments showed that the Hh signal reversed the effects of AHCY silence on inflammation and oxidative stress. This indicates that AHCY regulates oxidative stress *via* the Hh signaling pathway. This study verified the targeting relationship of AHCY with inflammation, oxidative stress and Hh signaling pathway. While WMP inhibited AHCY. Therefore, it can be concluded that WMP inhibits CAC inflammation and oxidative stress by targeting the regulation of AHCY-mediated Hh signaling pathway.

**Huangqin decoction (HQD)**, originating from *Shang-han-lun*, consists of 4 herbs, including *Scutellaria baicalensis Georgi* [Lamiaceae; Scutellariae radix] and *Paeonia lactiflora Pall.* [Paeoniaceae; Paeoniae radix alba]; it is the representative prescription for the treatment of heat dysentery. Chen et al. (Chen et al., 2016) developed four ways to prepare HQD through two different combination modes of water extraction/ethanol extraction and mixed extraction/separate extraction. Positive drug control group was not set. Four kinds of HQD intervened DSS-induced acute UC mice for 10 days, and HQD-1 (an aqueous extract decocted after the mixture of four herbal medicines) was screened as the optimal preparation method. Subsequent studies indicate that HQD-1 (9.1 g/kg) administered orally for 30 days can suppress TNF-α, IL-1β, and IL-6 in mice with chronic colitis, reduce the levels of the oxidative stress indicators MDA, 8-oxonine, and nitrotyrosine, stimulate SOD activity, and exert anti-inflammatory and antioxidative stress effects. In the CAC model, HQD-1 (9.1 g/kg) administered orally for 30 days inhibited the occurrence and growth of tumors and demonstrated anti-inflammatory and antioxidative stress effects. No concentration gradient for HQD was established in this study, so a dose-effect relationship for HQD could not be established. HQD can prevent and treat CAC, and has the effects of anti-inflammation and anti-oxidation, but the correlation between them cannot be clarified yet.

### 3.2 Induction of epithelial apoptosis

As mentioned above, the absence of p53 mutation function during the carcinogenesis of IBD is important and occurs early in the inflammation-cancer sequence. Research has shown that it even occurs before dysplasia (Beaugerie and Itzkowitz, 2015). p53 is an extremely critical tumor suppressor that integrates various stress signals to play an antitumor role, i.e., activating apoptosis being the most important (Güllülü et al., 2021). Under various stress stimuli in the inflammatory microenvironment, programmed cell death occurs in intestinal epithelial cells to ensure that they preserve normal physiological balance and prevent tumor formation; stress-induced apoptosis is mediated by p53 and B-cell lymphoma (Bcl) family proteins (Watson and Pritchard, 2000). Bcl family proteins, including Bcl-2, Bcl-xL, and Bax, mediate endogenous apoptosis (Yang et al., 2009) and are linked to multiple signaling pathways in the IBD-CAC process. Several classical signaling pathways, including the STAT3, NF-κB and Wnt pathways, have been majorly involved in the study of TCM formulas.

**Huangqi Baizhu decoction (HBD)**, derived from *Su-wen-bing-ji-qi-yi-bao-ming-ji*, comprises *Astragalus mongholicus Bunge* [Fabaceae; Astragali radix] and *Atractylodes lancea* (Thunb.) DC. [Asteraceae; Atractylodis rhizoma]. It is recorded in the original book used against dysentery; at present, it is often added to the basic herbs for dysentery treatment. Deng et al. (Deng et al., 2019a) intervened AOM/DSS induced mice with different doses of HBD aqueous extract (12, 24 g/kg) for 6 weeks. SASP (0.6 g/kg) was used as the positive control. The results showed that both HBD at 12 g/kg and SASP could effectively reduce the number and volume of tumors as well as the levels of TNF-α and IL-1β. Furthermore, HBD significantly downregulated the expression of IL-6/STAT3/Survivin/Cyclin D1 signal-related proteins. *In vitro* experiments, Atractylenolide II (ATR II) + Astragaloside (AST), the core component of HBQ, significantly inhibited the proliferation of CRC cells, as well as the IL-6-induced activation of the STAT3 signaling pathway and the expressions of Cyclin D1 and Survivin. The results of this study shows that HBD can inhibit tumor proliferation and reduce the expression of pro-inflammatory factors and anti-apoptotic proteins. Due to the lack of target verification, the molecular mechanism of HBD cannot be revealed yet.

**Changqing formula (CQF)** is an empirical prescription in TCM that is composed of 10 TCM herbs, including *Cuscuta australis* R. Br. [Convolvulaceae; Cuscutae semen] and *Epimedium brevicornu Maxim.* [Berberidaceae; Epimedii folium]. Luo et al. (2022) analyzed the blood absorbed components of CQF after gavage with network pharmacology and discovered that apoptosis, erythroblastosis oncogene B (ErbB) signaling and IL-17 signaling might take effect in the treatment of CRC with CQF. AOM/DSS induced mice were subsequently treated with 6.25 g/kg and 25 g/kg aqueous extracts of CQF for 12 weeks. Positive control group was not set. Consequently, CQF at 6.25 g/kg significantly suppressed tumor progression and promoted apoptosis. Transcriptomics revealed that after intervention with CAC, CQF primarily regulated IL-17, IL-6, NF-κB, and the apoptotic pathways. In addition, bioinformatics analysis indicated that metalloproteinase-9 (MMP-9) was involved in the occurrence and progression of CRC. The inhibitory effects of CQF on IL-17A, IL-6, NF-κB, and STAT3 were verified in subsequent experiments. Meanwhile, CQF also downregulated MMP-9, the anti-apoptotic protein Bcl-2, and upregulated the pro-apoptotic protein, Bax. This study indicates that CQF can promote apoptosis, inhibit tumor proliferation, and interfere with a variety of signaling molecules.

**Fengliao Changweikang (FLCWK)** is a Chinese patent medicine comprising 2 TCM herbs, i.e., *Daphniphyllum calycinum Benth.* [Daphniphyllaceae] and *Persicaria hydropiper* (L.) Delarbre [Polygonaceae]. FLCWK is used to treat gastrointestinal diseases including enteritis or diarrhea. Zhong et al. (2020) treated AOM/DSS-induced mice jointly with intragastric administration of different doses of FLCWK (4, 6, and 9 g/kg) and intravenous injection of 5- fluorouracil (5-FU, 24 mg/kg). The protocol in the paper only stated that FLCWK would be administered from the 49th day of the experimental cycle (72 days in total). However, the duration of administration was not specified. The result showed that unlike 5-FU alone, the combination of the two could improve the mouse survival rate, delay tumor proliferation, and reduce inflammatory infiltration in a dose-dependent manner. At the same time, the combined treatment demonstrated a better inhibitory effect on the IL-6/STAT3 signaling pathway, Bcl-2, and Cyclin D1 than 5-FU. *In vitro*, FLCWK at different intervention concentrations (5, 20, and 80 μg/mL) enhanced the proliferation inhibition and apoptosis of colon cancer cells driven by 5-FU (15 μM) in a dose-dependent manner. The combination of the two inhibited IL-6- stimulated STAT3 activation. This indicates that FLCWK cooperates with 5-FU to promote apoptosis and delay the carcinogenesis of colitis. There is no separate FLCWK group in this study, so it is difficult to determine the therapeutic effect of FLCWK on CAC without 5-FU.

**Compound sophorae decoction (CSD)**, an empirical formula made up of 6 TCM herbs, including *Sophora flavescens Aiton* [Fabaceae; Sophorae flavescentis radix] and *Sanguisorba officinalis L.* [Rosaceae; Sanguisorbae radix]; CSD has been widely used for UC in China. Deng et al. (Deng et al., 2019b) treated AOM/DSS induced mice by gavage with CSD aqueous extract (1.076 g/ml). Respectively during free drinking of DSS. The amount administered was fixed at 0.1614 g/d. No concentration gradient was set and there was no positive control drug. They discovered that the treatment delayed colitis symptoms in CAC mice and improved the extent of colon shortening and malignant hyperplasia. At the same time, CSD administration remarkably suppressed proinflammatory factors including TNF-α, NF-κB, IL-6, STAT3, and IL-17, as well as cyclooxygenase-2(COX-2) and inducible nitric oxide synthase (iNOS). CSD administration significantly upregulated caspase-3 and caspase-9 and downregulated Bcl-2 levels, confirming that it can promote apoptosis. Transmission electron microscopy (TEM) showed that inhibition of mitosis was observed in CAC mice dosed with CSD, consistent with pro-apoptotic effects. This indicates that CSD can promote apoptosis and prevent and treat CAC. The design of this study was not rigorous enough, for example, the concentration gradient was not set, and the drug dose was not calculated according to the body weight, which affected the reliability of experimental results.

**Yipi Yangwei decoction (YYD)**, an in-hospital preparation for the treatment of CRC in China Hospital, was prepared from 10 TCM herbs, including *Codonopsis pilosula* (Franch.) Nannf. [Campanulaceae; Codonopsis radix] and *Prunus mume* (Siebold) Siebold and Zucc. [Rosaceae;Linderae radix]. Lou et al. (2018) intervened AOM/DSS induced mice with different doses of YYD aqueous extract (4.29, 17.16 g/kg) for 11 weeks. Positive control group was not set. The results showed that YYD could delay the malignant proliferation of colon tissue in a dose-dependent manner. TEM showed sparsely arranged microvilli, swollen organelles, and damaged tight junctions in the colon mucosa, which were reversed following YYD administration. In addition, YYD administration downregulated the proinflammatory cytokines interferon (IFN)-γ and IL-12, upregulated the expression of the anti-inflammatory factor IL-4, and inhibited the activation of toll-like receptor 4/mitogen-activated protein kinase (TLR4/MAPK) and downstream NF-κB, thereby inhibiting proliferating cell nuclear antigen (PCNA), COX-2 and Bcl-xL, closely related to malignant inflammation and apoptosis of cells. It can be concluded that YYD promotes apoptosis, inhibits malignant inflammation, and delays the proliferation of CAC. The correlation between histological improvement and signaling pathway molecules remains to be confirmed.

**Huangqin Tang (HQT)** has a similar composition to HQD. Ma et al. (Ma et al., 2022) treated AOM/DSS-induced mice for 7 weeks by gavage with HQT aqueous extract (20 g/kg). Positive control was not set. The study found that HQT effectively ameliorated symptoms including diarrhea, empyema, hematochezia, and weight loss as well as histological damage to the colon in CAC mice and downregulated the levels of IL-1β, IL-6, and TNF-α. Proteomic analysis showed that HQT regulates extracellular matrix (ECM)-related adhesion molecules, tumor cell differentiation, metastasis, and apoptosis and anchored apoptosis-related as well as Wnt/β-catenin-related proteins, which might be the key targets of HQT for CAC. Subsequent experiments confirmed that HQT downregulated cyclin d1(Ccnd1), cyclin-dependent kinase 4(Cdk4), Wnt3a and catenin beta 1(Ctnnb1) and upregulated the expression of glycogen synthase kinase-3β(Gsk-3β) and cadherin 1(Cdh1). The Cdh1 gene encodes the E-cadherin protein, and its loss characterizes the occurrence of EMT and is related to tumor metastasis and invasion (Onder et al., 2008). This indicates that HQT may suppress the development of IBD-CAC by promoting apoptosis and inhibiting EMT *via* the Wnt/β-catenin pathway. The concentration gradient was not set in this study and therefore the dose-effect relationship for HQT is unknown.

### 3.3 Regulation of the tumor microenvironment

As a tumor mediated by the accumulation of gene mutations due to continuous inflammatory stimulation, CAC is closely associated with changes in TME in which epithelial cells live (Fantini et al., 2020). The TME refers to the non-cancer cell components in the extracellular matrix (ECM) where the tumor is located and the mutual crosstalk between them (Jarosz-Biej et al., 2019). Its interaction with cancer cells determines the ensuing development trends of the tumor (Jarosz-Biej et al., 2019). As an essential part of the tumor matrix, immune cells occupy a central position in the TME. However, they are not all health-friendly to the body. Cell types, including tumor-associated macrophages (TAMs) of the M2 phenotype (Hardbower et al., 2017), tumor-associated neutrophils (TANs) of the N2 phenotype (Zheng et al., 2022), myeloid-derived suppressor cells (MDSCs) (Wang et al., 2020), and specific types of regulatory T-cells (Tregs) (Rizzo et al., 2018), can suppress the antitumor immune response and promote angiogenesis to create a hypoxic microenvironment, helping tumor cells in immune escape. At the same time, various cytokines and chemokines secreted by immune cells in the TME, including transforming growth factor beta (TGF-β), IL-6, and hepatocyte growth factor (HGF), can induce EMT in adjacent cells in a paracrine manner (Dongre and Weinberg, 2019). Noteworthy, in CAC, epithelial cells are under continuous malignant inflammatory stimulation, and inflammatory signaling pathways are in a long-term activation state. While recruiting immune cells, signaling pathways including hypoxia in the matrix and EMT are also activated, and the crosstalk between them accelerates the carcinogenesis of IBD-CAC (D’Ignazio et al., 2017).

#### 3.3.1 Regulation of tumor immune microenvironment

**Huang Qin decoction (HQD)** has a similar composition as previously mentioned. Pan et al. (2022) treated AOM/DSS induced mice with different doses of HQD aqueous extract (0.5, 1.0, and 1.5 g/kg) for 7 weeks. Aspirin (40 mg/kg) was used as the positive control drug. HQD administration at a minimum dose of 1.0 g/kg was found to inhibit intestinal tumor growth in a dose-dependent manner. Aspirin also had a tumor suppressor effect similar to that of HQD at 1.5 g/kg. HQD repaired the intestinal mucosal barrier by upregulating Occludin and zona occludens-1 (ZO-1). After HQD treatment, neutrophils and CD11b^+^Ly6C^+^ monocytes increased in the mice, whereas the lymphocytes increased. In addition, HQD downregulated the expression of programmed cell death protein 1 (PD-1) in CD8^+^T-cells. PD-1 can induce, establish and maintain immune tolerance in the TME and help cancer achieve immune escape (Han et al., 2020), an important immune checkpoint (Xu-Monette et al., 2018). Therefore, HQD enhanced immune surveillance of CD8^+^ T-cell. Network pharmacology analysis revealed that peptidyl arginine deaminase 4 (PAD4) might be the key target of HQD. PAD4 is activated in neutrophils and can mediate the formation of neutrophil extracellular traps (NETs) (Hawez et al., 2019). Recent studies indicate that the formation of NETs in IBD increases significantly (Mortensen et al., 2022) and is associated to the occurrence of CRC (Chen et al., 2022). NETs markers decreased after HQD treatment. This indicates that HQD can slow down intestinal inflammation, inhibit tumor proliferation and reduce NETs aggregation, which has a potential regulatory effect on tumor immune microenvironment.

**Xian-Lian-Jie-Du decoction (XLJDD)** is an empirical prescription for the treatment of CRC developed by Qihuang scholar Professor Cheng Haibo. It comprises 4 herbal medicines, including *Agrimonia pilosa Ledeb.* [Rosaceae; Agrimoniae herba] and *Coptis chinensis Franch.* [Ranunculaceae; Coptidis rhizoma]. Yu et al. (Yu et al., 2022) intervened AOM/DSS and high-fat diet-induced mice for 17 weeks by gavage with XLJDD aqueous extract (12.9 g/kg, the best intervention dose determined in previous studies) and its five main components, respectively. Folic Acid was used as the positive control drug (the dose is unknown). They found that XLJDD exerted better inhibition on tumors than every single group. RNA-seq analysis of tumor tissues in each group revealed that XLJDD treatment was associated with inflammatory factor reactions and cell death-related pathways, and IFN-related pathways were significantly enriched. IFN can induce tumor cell death (Chawla-Sarkar et al., 2003). Subsequent experiments confirmed the occurrence of apoptosis and revealed that XLJDD significantly improved the proportion of B-cell clusters in the tumor immune microenvironment, which was associated with the ferroptosis pathway. Key genes, including Mfsd2a and Ccdc85c in the XLJD group, were identified through multi-group analysis. The former was up-regulated in CAC tissue and significantly down-regulated after XLJDD intervention, whereas the latter showed the opposite trend. Based on previous studies, Mfsd2a has a protective effect on colitis (Ungaro et al., 2017), whereas Ccdc85c is related to malignant proliferation (Tanaka et al., 2017). This study shows that XLJDD can promote cell death, regulate immune microenvironment and prevent CAC. Although the key gene of XLJDD in treating CAC has been obtained through multi-omics screening, its molecular mechanism is still unclear due to the lack of target verification experiments.

#### 3.3.2 Intervention with crosstalk among inflammatory signals, immune cells, and hypoxia

**Yi-Yi-Fu-Zi-Bai-Jiang-San (YYFZBJS)** is derived from *Jin-gui-yao-lue* and comprises 3 herbs, including *Coix lacryma-jobi* var. ma-yuen (Rom.Caill.) Stapf [Poaceae; Coicis semen] and *Aconitum carmichaelii Debeaux* [Ranunculaceae; Aconiti lateralis radix praeparata], which are recorded in medical books for treatment of intestinal carbuncles. Zhang et al. (Zhang et al., 2022) treated AOM/DSS induced mice with different doses of YYFZBJS aqueous extract (3.825, 15.3 g/kg) for 12 weeks. Aspirin (30 mg/kg) was used as the positive control. They discovered that YYFZBJS with two doses significantly inhibits the inflammation-to-cancer transformation of IBD and regulates the mucosal immune response. No difference in hepatorenal toxicity was noted between treatment groups, indicating that the herbal doses were safe. RNA-seq analysis revealed that genes related to the hypoxia-inducible factor (HIF) -1α signaling pathway were significantly downregulated in the YYFZBJS high-dose group, and the phenotypic prediction was closely associated with Treg differentiation. Subsequent experiments confirmed that YYFZBJS significantly down-regulated the level of HIF-1α and increased the infiltration of CD4^+^ CD25^+^ cells in the tumor. *In vitro* experiments confirmed that the level of HIF-1α expressed by Tregs was substantially increased in a hypoxic environment. Additionally, HIF-1α overexpression reversed the inhibitory effect of Tregs on the proliferation of colon cancer cells, with no change in the expression of forkhead box protein P3 (Foxp3^+^) Tregs. Of note, Foxp3^+^ is a marker of naturally occurring Tregs (nTregs) (Zhang and Zhou, 2019). Therefore, HIF-1α may regulate IBD-CAC transformation by targeting peripheral Tregs (pTregs) rather than the polarization of nTregs. Subsequently, CRC cell proliferation was improved through the addition of HIF-1α-transfected Tregs in a co-culture system of CRC cells and normal epithelial cells. In contrast, the YYFZBJS extract delayed CRC cell proliferation, down-regulated phosphatidylinositide 3-kinases (PI3K), p-STAT3, and p-NF-κB expressions, closely related to malignant proliferation. Further *in vivo* verification in a Treg-depleted mouse model revealed that the antitumor effect of YYFZBJS was suppressed by the absence of Tregs. The study is relatively rigorous in design. It indicates that YYFZBJS improves the tumor immune microenvironment *via* HIF-1α-induced Treg polarization, thereby preventing the occurrence and development of CAC.

#### 3.3.3 Intervention with crosstalk among inflammatory signals, immune cells, and EMT

**Shenling Baizhu San (SBS)** is derived from *Tai-ping-hui-min-he-ji-ju-fang* and consists of 10 herbs, including *Panax ginseng* C.A.Mey. [Araliaceae; Ginseng radix et rhizoma] and *Poria cocos* (Schw.) Wolf [Polyporaceae; Poria]. SBS is a commonly used prescription for the treatment of chronic diarrhea. Lin et al. (2015) intervened AOM/DSS induced mice by gavage with aqueous extract of SBS (7.28 g/kg). It could not find the intervention time of SBS clearly in the paper. Positive control was not set. They found that it exerted anti-inflammatory and anticancer effects and could reduce the infiltration of CD11b^+^Ly6C^+^ cells and serological level of MDSCs effector molecule TGF-β1. CD11b^+^Ly6C^+^ is a phenotype of MDSCs with immunosuppressive effects (Zhao et al., 2016), producing chemokines such as TGF-β1 to induce EMT (Taki et al., 2021). SBS increased E-cadherin, an EMT epithelial phenotype; reduced N-cadherin, fibronectin, vimentin and the transcription factor Snail, as well as Wnt5a; upregulated the expression of axis inhibition (Axin), dishevelled 2 (Dvl2) and p-GSK-3β. SBS and its component Atractylenolide-1 (AT1) inhibit the proliferation of colon cancer cells in a dose-dependent manner *in vitro*. In addition, SBS reduced TGF-β1-induced activation of CRC cell matrix signal Wnt5a and reversed the down-regulation of E-cadherin. In this study, drug concentration gradient and positive control drug were not set, and the intervention time of SBS was not clear, which reduced the reliability of conclusions.

**Shaoyao decoction (SYD)** has the same composition as described before. Lin et al. (2014) intervened AOM/DSS induced mice by gavage with SYD aqueous extract (7.12 g/kg). We were unable to identify the intervention time of SYD in the thesis. No positive controls were set for this study. SYD effectively improved CAC symptoms and reduced tumor incidence, tumor burden, and expression of oncogenic proteins. *In vitro* experiments have also confirmed that both SYD and its component paeonol can suppress the proliferation of CRC cells and the tumor proliferation-related proteins PCNA, β-catenin, and p53 in a dose-dependent manner. This indicates that SBS may inhibit the malignant progression of IBD-CAC. In animal experiments, SYD repressed the EMT transcription factor Snail and regulated the expression levels of downstream EMT-related proteins including E-cadherin, N-cadherin, and Vimentin. SYD downregulated the levels of the serum inflammatory factors IL-1β, IL-6, TNF-α, expression of NF-κB p65, and the TAMs marker F4/80+. This suggests that SYD can prevent and treat CAC, inhibit the infiltration of TAMs and the occurrence of EMT induced by Snail. There was no concentration gradient of SYD in this study, and the research design was not rigorous enough.

### 3.4 Regulation of the intestinal microbiota and its metabolites

The first line of protection against intestinal pathogens is provided by intestinal epithelial cells (Shawki and McCole, 2017). IBD is a disease of dysfunction barrier. Defects in the intestinal mucosal barrier increase the susceptibility of diseased tissue to intestinal cavity bacteria and their metabolites, hence making the intestinal flora occupy an important position in inflammation and immune microenvironment modeling. Accumulating studies indicate that certain specific bacteria and their derived molecules promote the development of CAC (Cheng et al., 2020). *Fusobacterium nucleatum* can activate the EFGR signaling pathway to promote EMT (Yu et al., 2020), and develop a pro-inflammatory microenvironment conducive to the progression of colorectal tumors by recruiting tumor-infiltrating immune cells (Kostic et al., 2013). *Enterotoxigenic Bacteroides fragilis* directly targeting intestinal epithelial cells to aggravate the pro-cancer inflammatory cascade (Chung et al., 2018). Lipopolysaccharide (LPS) produced by Gram-negative bacteria can induce pyroptosis of inflammatory cells (Kayagaki et al., 2015). Beneficial bacteria including *Bifidobacterium* (Kim et al., 2010) and *Lactobacillus* (Mann et al., 2013) promote immune regulation with a therapeutic effect on CAC. In addition, bile acids and short-chain fatty acids (SCFAs), metabolites of intestinal flora regulate the pathogenesis, prophylaxis, and treatment of CAC (Zeng et al., 2019). Specifically, different SCFAs are produced by the metabolism of different flora. Among them, butyrate is an important substance that provides energy for colonic epithelial cells, maintains cell homeostasis (Donohoe et al., 2011), can regulate T-cell activation (Kespohl et al., 2017), and suppress cancer cell invasion (Zeng and Briske-Anderson, 2005). Therefore, the butyric acid-producing bacteria in CAC are important.

#### 3.4.1 Intervention of crosstalk between intestinal microbiota and inflammatory signals

**Huoxiang Zhengqi (HXZQ)**, derived from *Tai-ping-hui-min-he-ji-ju-fang*, comprise 13 herbs, including *Atractylodes macrocephala Koidz.* [Asteraceae; Atractylodis macrocephalae rhizoma] and *Citrus × aurantium* f. deliciosa (Ten.) M. Hiroe [Rutaceae; Citri reticulatae pericarpium]. HXZQ has been made into Chinese patent medicine and is broadly applied for the treatment of diarrhea. Dong et al. (2022) treated AOM/DSS induced mice with different doses of HXZQ (0.45, 1.35 g/kg) for 6 weeks. Positive control was not set. They found that HXZQ could suppress genes and proteins related to the Nrf2/NF-κB/NLRP3 signaling pathway, release of inflammatory factors and the expression of oxidative stress markers and inhibit the occurrence and development of CAC. The dosage of 1.35 g/kg was better than 0.45 g/kg. At the phylum level, HXZQ increased *Bacteroidetes* and decreased the proportion of *Firmicutes* unlike in the model group. At the genus level, HXZQ reduced the abundance of *Candidatus Arthromitus, Dorea,* and other genera; it also increased the abundance of butyric acid-producing bacteria including *Anaerotruncus, Prevotella, Butyricimonas,* and other genera. Butyrate is a four-carbon SCFAs produced by the fermentation of dietary fiber by specific flora with anti-inflammatory properties that can improve intestinal mucosal immunity and barrier function (Liu et al., 2018). This indicates that HXZQ can inhibit Nrf2/NF-κB/NLRP3 signaling pathway, resist oxidative stress and positively regulate intestinal microbiota. This may be the potential mechanism of HXZQ in preventing and treating CAC.

**Chang-wei-qing (CWQ)** is a prescription developed by Professor Fan Zhongze, a famous TCM expert, for treating malignant tumors of the digestive tract. It comprises 8 herbs, including *Astragalus mongholicus Bunge* [Fabaceae; Astragali radix] and *Atractylodes lancea* (Thunb.) DC. [Asteraceae; Atractylodis rhizoma]. Wan et al. (2019) intervened AOM/DSS induced mice with different intervention doses of CWQ (5, 10 mg/kg) for 89 days. Bifico (4.2 g/kg) was used as the positive control. Bifico or CWQ at different doses can restore colon length, and minimize inflammation and the number and volume of tumors. CWQ demonstrated a dose-dependent effect. After treatment with Bif or CWQ, no statistical difference was noted in the number of total bacteria in the mouse feces, unlike the model group, whereas the levels of *Faecalibacterium prausnitzii* (*F. prausnitzii*) and bacterial metabolite β-glucuronidase (BGUSs) were down-regulated compared to the model group. BGUSs are involved in the catabolism of carbohydrates. They are known to prevent elimination of certain carcinogens from the digestive tract of the host, which increases the risk of CRC (Zhang et al., 2019a). It has been shown that Bif or CWQ reduces the levels of d-lactose and LPS in peripheral blood, both of which are metabolites of intestinal microflora. Moreover, Bif or CWQ can down-regulate TLR4, NF-κB p65, P-STAT3, and Bcl-2 expression levels. Therefore, CWQ might inhibit CAC progression by regulating the gut microflora and their metabolites such as BGUSs and LPS. Due to the lack of target verification, it is impossible to draw exact conclusions about the molecular mechanism of CWQ. Unlike many studies, *F. prausnitzii is a* well-known butyric acid-producing bacteria of the *Firmicutes* phylum and is a protective factor for IBD and CRC (Lopez-Siles et al., 2017). Here, *F. prausnitzii* abundance was however downregulated with the improvement of the disease after treatment. We believe that this requires further study and explanation by the researchers.

**San-Wu-Huang-Qin decoction (SWHQ)** is derived from *Jin-gui-yao-lue* comprising 3 drugs, i.e., *Scutellaria baicalensis Georgi* [Lamiaceae; Scutellariae radix] and *Sophora flavescens Aiton* [Fabaceae; Sophorae flavescentis radix], often used for the treatment of CRC. Zhou et al. (2022) intervened AOM/DSS induced mice with SWHQ aqueous extracts (1.82, 3.64 g/kg) for 11 weeks. Aspirin (1.4 mg/kg) was used as the positive control. The results showed that SWHQ or Aspirin could effectively reduce the tumor burden. SWHQ was dose-dependent. 7.28 g/kg SWHQ was superior to Aspirin. The result indicated that SWHQ exhibits a significant antitumor effect. TEM and detection of tight junction proteins revealed that SWHQ restored the integrity of the intestinal mucosal barrier. 16S rRNA analysis of feces revealed that SWHQ could reverse the imbalance of *Firmicutes* and *Bacteroides* caused by the AOM/DSS model, upregulate the abundance of the beneficial bacteria *Akkermansia* and *Faecalibaclum,* and reduce the abundance of the pathogenic bacteria *Bacteroides, Entero Coccus* and *Escherichia-Shigella*. PICRUSt analysis showed a significant reduction in bacterial LPS synthesis in the SWHQ group. The intestinal microbiota of the model and SWHQ groups were colonized in the sham-sterile model mice *via* fecal microbiota transplantation (FMT), and the findings revealed that the antitumor and mucosal protection effects of the FMT-SWHQ group were similar to that of the donor group. 16S rRNA also showed similar results to that of the donor group, indicating that changes in the intestinal flora of the donor were partially replicated in the receptor. Based on the analysis of intestinal microflora, targeting the intestinal microflora, particularly the LPS-producing flora, is an important mechanism of SWHQ against tumors. Of interest, both species identified by 16S rRNA are related to butyric acid in colitis. *Faecalibaclum* is a butyric acid-producing bacterium and has a negative correlation with the activity of UC (Machiels et al., 2014). *Akkermansia* has anti-inflammatory properties in DSS-induced colitis and is positively correlated with the concentration of butyric acid (Wan et al., 2021). Further experiments showed that both SWHQ and FMT-SWHQ significantly down-regulate TLR4/NF-κB activation and the expression of inflammatory factors in the mouse colon tissue. Taken together, this study was well designed, and verified that intestinal flora was the target of SWHQ in the intervention of CAC.

**Wu Mei Wan (WMW)** has a similar composition to WMP. Jiang et al. (2020) intervened AOM/DSS induced mice with WMW aqueous extract (5.8 g/kg) for 10 weeks. Positive control was not set. They discovered that WMW could significantly reduce AOM/DSS-induced weight loss, tumor number, and tumorigenic grade. Mechanistic studies have shown that WMW can reduce the expression of NF-kB, p65, P-STAT3, and IL-6 in colon tissue and downregulate the serum IL-6 level. Previous studies have shown that the activation of STAT3 and NF-κB signals is important to the development of CAC (Waldner and Neurath, 2015). Fecal 16S rDNA sequencing analysis showed that after WMW intervention, *Bacteroides* decreased and *Firmicutes* increased at the phylum level; *Bacteroides_S24-7_group* decreased, whereas *Lachnospiraceae* increased at the family level, unlike the model group. The role of *Bacteroidales_S24-7_group* has always been controversial. *Lachnospiraceae* is a typical obligate anaerobe-producing butyrate (Leth et al., 2022), promoting colonization resistance of the host against intestinal pathogens and is beneficial to the body (Sorbara et al., 2020). This study shows that WMW can regulate intestinal flora and inhibit the transformation of IBD from inflammation to cancer. Due to the lack of targeted verification, it cannot be explained that intestinal flora is the target of WMW in the treatment of CAC.

**Canmei formula (CMF)** was derived from *Ben-cao-gang-mu* and comprise 2 drugs, i.e., *Prunus mume* (Siebold) Siebold and Zucc. [Rosaceae; Mume fructus] and *Bombyx mori Linnaeus* [Bombycidae; Bombyx batryticatus]. CMF was used to treat hematochezia. Zhang et al. (Zhang et al., 2019b) used CMF ethanol extract (CMF-A 0.657 g/kg) and CMF aqueous extract (CMF-L 3.65 g/kg, CMF-H 7.3 g/kg) to treat AOM/DSS and high-fat-diet-induced C57BL/6 mice for 68 days, respectively. Aspirin (1.4 mg/kg) was used as the positive control. The results indicated that CMF observably suppressed the occurrence and growth of tumors in mice, whereas aspirin only inhibited the growth of large (d ≥ 3 mm) colon adenomas. In addition, the CMF-A, CMF-L/CMF-H, and aspirin groups significantly reduced the expression of IL-17C and NF-κB. The 16S rDNA results revealed a decrease in bacterial population diversity in the model group and a significant increase after treatment with CMF-A, whereas aspirin did not display this capacity. *Muribaculaceae, Bacteroides, and Preceptellaceae* were the most abundant flora at the family level. After treatment with CMF, the abundance of *Desulfovibrionaceae* was significantly reduced compared to the model. One study has shown that *Desulfovibrionaceae* is significantly enriched in mice fed with a high-fat diet (Zhang et al., 2021a) and that the high-fat diet can drive AOM/DSS-induced CAC (Choi and Snider, 2019), hence *Desulfovibrionaceae* may be the key driving CAC. After treatment with CMF-A, the levels of *Bacteroidaceae* and *Staphylococcaceae* significantly changed from family to genus, and *Turicibacter, Bacteroides, Bacteroidaceae, and Faecalibaculum* were significantly enriched at the genus level. Of interest, Zhong et al. (2015) used dietary fiber to regulate the intestinal flora of high-fat diet-fed rats, and the results showed that the abundance of *Turicibacter* positively correlated with butyric acid concentration. This is consistent with the modeling method and findings in this study. There is a likelihood that CMF positively regulates intestinal inflammation *via* flora and its metabolites butyric acid, which requires further experimental verification.

**Qingchang Wenzhong decoction (QCWZD)** was developed by Li and consists of 8 Chinese medicinal materials, including *Coptis chinensis Franch.* [Ranunculaceae; Coptidis rhizoma] and *Zingiber officinale Roscoe* [Zingiberaceae; Zingiberis rhizoma praeparatum]. QCWZD is used to treat UC (Xia et al., 2022). Ren et al. (2022) intervened AOM/DSS induced C57BL/6/6 mice with different doses of QCWZD (0.7, 1.4 g/kg) for 15 weeks. QCWZD at 1.4 g/kg was used to intervene in a high-fat diet combined with an Apc^min/+^ induced mouse model for 14 weeks. Positive control group was not set. The results indicated that QCWZD at 1.4 g/kg could significantly reduce the tumor burden of the two models, as well as improve the histological inflammatory injury and repair the intestinal mucosal barrier. QCWZD also suppressed the expression of two models pyroptosis-related proteins NLR pyrin domain containing 3 (NLRP3), gasdermin E (GDSME), high-mobility group box 1 protein (HMGB1), IL-1β and IL-18. The intestinal bacterial metabolites LPS and nigericin et al. can activate NLRP3, cut the scorch death-inducing protein GDSME, and promote the conversion of pro-IL-1β and pro- IL-18 into their active forms, resulting in pyroptosis of inflammatory cells (Wang et al., 2021a). Fecal 16S rRNA sequencing showed that QCWZD could significantly alter the overall composition of intestinal flora in the two model mice. In the AOM/DSS induction study group, *Desulfobacterota* was present only in the model group at the phylum level. At the genus level, the abundance of *Bacteroides*, *Colidextribacter*, and *Muribaculum* was significantly downregulated, whereas that of *Bosea*, *Caulobacter*, and *Ralstonia* was significantly upregulated compared to the model group. The high expression of *Muribaculum* is regarded as a potential marker of metabolic disorder of bile acids (BAs) (Marion et al., 2020). Secondary BAs, such as deoxycholic acid and lithocholic acid, are risk factors for IBD and CRC (Zeng et al., 2019). In the high-fat diet + Apc^min/+^ induction study group, the QCWZD high dose group up-regulated the abundance of *Desulfobacterota, Anaerotruncus,* and *Butyricoccus* at phylum level and down-regulated the abundance of *Erysipelotrichaceae_unclassified,* unlike the model group. The *Anaerotruncus and Butyricimonas* upregulated after treatment were all butyric acid-producing bacteria. Interestingly, previous studies indicate that a decrease in the abundance of *Desulfobacterota* is a common feature of acute and chronic disease stages in patients with UC (Zhu et al., 2022). *Desulfobacterota* was increased after AOM/DSS modeling, as well as significantly after high-dose QCWZD intervention in Apc^min/+^ mice, yielding a controversial finding on whether *Desulfobacterota* is beneficial or pathogenic. This difference may also be caused by tumors with different phenotypes caused by different modeling methods. Based on the above results, it can be concluded that QCWZD can treat CAC and small intestinal tumors. This effect may be achieved by mediating intestinal flora and cell death, but it needs more rigorous and perfect experimental verification.

#### 3.4.2 Intervention with crosstalk among intestinal microbiota, inflammatory signals, and immune cells

**Pai-Nong-San (PNS)** is derived from *Jin-gui-yao-lue* and consists of 3 herbal medicines, including *Citrus × aurantium* L. [Rutaceae; Aurantii fructus immaturus] and *Paeonia lactiflora Pall.* [Paeoniaceae; Paeoniae radix alba]. PNS is used for the treatment of carbuncles and pus. Zhang et al. (2021b) intervened AOM/DSS induced mice with different doses of PNS aqueous extract (0.8, 1.6, 3.2 g/kg) for 9 weeks. Aspirin (13 mg/kg) was used as the positive control drug. PNS at 0.8 g/kg was found to be the lowest dose effective in preventing tumorigenesis. PNS exhibited a dose-dependent behavior. PNS could effectively prevent tumor occurrence. Additionally, PNS and Aspirin up-regulated anti-inflammatory factors, down-regulated pro-inflammatory factors, and HIF-α levels. At the same time, the infiltration of CD4^+^ and CD8^+^T lymphocytes in colonic tissues was significantly reduced. The effect of PNS was dose-dependent. 16S rRNA sequencing results showed that at the phylum level, PNS treatment adjusted the modeling-induced abundance abnormalities of *Bacteroides, Proteobacteria,* and *Firmicutes* to normal levels, however, aspirin only corrected the abundance abnormalities of *Bacteroides.* At the genus level, PNS significantly increased the abundance of *Lactobacillus*. *Lactobacillus* is the most common probiotic genus in the human intestinal tract. It can effectively decompose sugars and produce lactic acid, enhance the intestinal antioxidant stress capacity, and inactivate carcinogenic factors (Eslami et al., 2019). *Lactobacillus* positively correlated with a butyric acid concentration in UC rats (Wang et al., 2022b). Clustering analysis showed that the PNS group separated from the CAC group and was close to the normal group, indicating that PNS had a positive regulatory effect on the intestinal flora. In contrast with the model, PNS suppressed the expression of p-GSK-3β, β-catenin, and c-Myc, indicating that PNS could suppress the activation of the Wnt/β-catenin signaling pathway. This study shows that PNS might prevent the progression of IBD-CAC by inhibiting the Wnt/β-catenin pathway, CD4^+^, and CD8^+^ T-lymphocyte infiltration, as well as positively regulating the intestinal flora. Certainly, this also needs further targeted verification.

**Yi-Yi-Fu-Zi-Bai-Jiang-San (YYFZBJS)** is the same composition as described before. Chai et al. (2021) intervened AOM/DSS induced mice with YYFZBJS ethanol extract (3.825, 7.65, and 15.3 g/kg) for 9 weeks at different administration doses. Aspirin (30 mg/kg) was used as the positive control drug. They discovered that YYFZBJS significantly reduced the occurrence of CAC in a dose-dependent manner. 16S rRNA sequencing showed that YYFZBJS could enhance the α diversity of the intestinal flora. Unlike the CAC model, YYFZBJS increased the abundance of *Lactobacillus, Ruminococcaceae,* and *Clostridium*, but decreased the abundance of *enterotoxigenic Bacteroides fragilis (ETBF)* in a dose-dependent manner. Interestingly, all three added bacteria could produce butyric acid (Guo et al., 2022; Ávila et al., 2023). *Ruminococcaceae* can regulate secondary BAs, promote the malignant progress of inflammation (Sinha et al., 2020), and may play a role in anti-tumor immunity (Peng et al., 2020). The research of Zhong et al. (2015) shows that Clostridium is significantly related to the metabolite succinic acid, which is an index to inhibit EMT of CRC stem cells (Terasaki et al., 2018). AOM/DSS-induced sterile mice were infected with ETBF, and ETBF infection promoted tumor proliferation. Subsequently, Ray Biotech revealed that *ETBF* activated the expression of M2-type macrophage cytokines in sterile mice. In contrast with the *ETBF* group, the number of adenomas was significantly reduced after intervention with YYFZBJS, and the expression of M2 markers was downregulated, whereas the secretion of M1-related protein was upregulated, suggesting that YYFZBJS might prevent and treat CAC by regulating macrophage polarization with *ETBF*. *In vitro* assay, ETBF was co-cultured with colon cancer cells and significantly up-regulated JNK, STAT3, NF-κB, and Arg-1. M2-type BMDMs were isolated from mice treated with ETBF and ETBF + YYFZBJS, respectively, to interfere with colon cancer cells *in vitro*. Consequently, BMDMs in the ETBF + YYFZBJS group suppressed the proliferation of colon cancer cells and reduced the expression of p-STAT3, MMP-2, MMP-9, cyclinD1, and c-Met; conversely, STAT3 was activated in the ETBF group. This study was designed in detail, and the results showed that the mechanism of YYFZBJS treating CAC might be achieved by regulating intestinal flora and reducing ETBF-mediated polarization of M2 macrophages.

**Sini decoction (SND),** originating from *Shang-han-lun*, comprises 3 herbal medicines, including *Aconitum carmichaelii Debeaux* [Ranunculaceae; Aconiti lateralis radix praeparata] and *Glycyrrhiza uralensis* Fisch. ex DC. [Fabaceae; Glycyrrhizae radix et rhizoma], and is suitable for the treatment of yang deficiency syndrome in TCM. Wang et al. (2021b) intervened AOM/DSS induced mice with SND aqueous extract (3.5 g/kg) for 90 days. Positive control drug was not set. They discovered that SND could effectively prevent and treat CAC. Immunohistochemical results showed that SND increases CD8^+^ T-cells in mouse colon tissue and decreases the infiltration of CD4^+^ T-cells. Meanwhile, SND increased Occludin and downregulated the expression of IL-6, IL-17, TNF-α, IFN-γ, and CRC markers. 16S rRNA sequencing showed that, unlike the model group, SND reduced the abundance of *Bacteroidetes* and increased the abundance of *Firmicutes* at the phylum level. At the genus level, *Lactobacillus gasseri and Bacillus coagulans* were significantly enriched in the SND group, whereas *Bacteroides fragilis* was significantly enriched in the CAC group. One study found that the abundance of *Bacteroides fragilis* increases in patients with colonic precancerous lesions and early cancer, suggesting that it may be a risk factor and biomarker for CRC (Zamani et al., 2019). After SND treatment, the abundance of *Bacteroides fragilis* decreased, whereas the abundance of beneficial bacteria including *Bacillus coagulans, Akkermansia muciniphila, and Bifidobacterium* increased. *Bacillus coagulans* is the producer of hyperbutyrate (Shi et al., 2022), and *Lactobacillus gasseri* and *Akkermansia muciniphila* also belong to the butyric acid-producing bacteria. *Bifidobacterium* is a lactic acid-producing probiotic that can also work with other microorganisms to increase the rate of butyrate production (Rivière et al., 2015). Sivan A et al. (Sivan et al., 2015) have found that oral administration of *Bifidobacterium* can promote the anti-tumor immunity of tumor patients. One study has shown that the abundance of *Lactobacillus, Bifidobacterium,* and *Akkermansia* could be increased following *Lactobacillus gasseri* intervention in AOM/DSS induced mice (Oh et al., 2020). The Result was similar to those in this study, indicating the protective effect of *Lactobacillus gasseri* on CAC. This study shows that SND may inhibit the occurrence and development of CAC by mediating the crosstalk between some specific flora such as *Bacteroides fragilis* and immune cells CD4^+^ CD8^+^. Since there is no drug concentration gradient, the dose-effect relationship of SND cannot be obtained.

## 4 Conclusion and perspective

The incidence and prevalence of IBD in China and other Asian countries have been increasing in the past 30 years, causing heavy disease and economic burden (Shao et al., 2022). IBD can develop into CAC *via* the sequence “chronic inflammatory response-low grade dysplasia-high grade dysplasia-cancer”, one of the most fatal complications of IBD. There are many adverse reactions in drug treatment of IBD and CAC (Kumar et al., 2012). At present, No effective drug preventing the conversion of IBD to CAC remains unavailable. In recent years, TCM has demonstrated significant potential in the prevention and treatment of CAC. TCM formula is the major carrier for the treatment of diseases in TCM and studies on the prevention and treatment of CAC with TCM formulas have emerged. Based on the unique pathogenesis of CAC, we reviewed the research progress in the prevention and treatment of CAC with TCM formulas.

We found eighteen TCM formulas, exerting a therapeutic effect on CAC (Table 1). Among them, the preventive and therapeutic effects of SYD, HQD/HQT, WMP/WMW, and YYFZBJS on CAC have been repeatedly confirmed by different research teams, indicating that they are more likely to be prescriptions with anticancer potential. Additionally, TCM formulas are characterized by multiple targets and pathways. For instance, SYD not only inhibits inflammation and oxidative stress *via* Nrf2/ARE signaling pathway (Wang et al., 2019), but also inhibits NF-κB activation as well as TAM, and EMT infiltration (Lin et al., 2014). Furthermore, WMP/WMW not only suppresses the AHCY-mediated Hh signaling pathway and oxidative stress in inflammation (Wang et al., 2022a) but also mediates the positive regulation of intestinal flora composition by NF-κB and IL-6/STAT3 (Jiang et al., 2020). Generally, TCM formulas for preventing and treating CAC majorly regulate the biological processes, including inflammation-mediated oxidative stress, apoptosis, TME, and intestinal microecology imbalance (Figure 1). We found that the regulation of TCM formula on the immune microenvironment and intestinal flora is a current research focus, involving the complex crosstalk between the intestinal flora, immune cells, EMT, and hypoxia. Specifically, we found that the abundance of butyric acid-producing bacteria including *Faecalibaclum, Lachnospiraceae, Akkermansia, and Prevotella* significantly increased after treatment. Butyrate supports the proliferation of healthy colonic crypts and preserves colonic homeostasis, inducing tumor cell apoptosis with an anti-colitis effect (Hanus et al., 2021). Therefore, increasing the abundance of butyric acid-producing bacteria may be a target of TCM formulas. Non-etheless, the majority of studies we included did not detect the metabolism of targeted SCFAs or analyze the correlation between SCFAs and microbial community diversity. Therefore, it cannot be directly established whether these TCM formulas can improve the yield of butyric acid. In addition, we found the potential of TCM formulas in the regulation of programmed cell death (PCD). PCD primarily includes apoptosis, pyroptosis, ferroptosis, autophagy, etc., referring to the process that cells actively die after receiving certain signals or stimuli to maintain the internal environment’s steady state (Li et al., 2022). In the studies, we included, HBD, HQD, CSD, and other prescriptions significantly promoted the apoptosis of colon cancer cells, effectively inhibiting the proliferation of cancer cells out of control. Among them, XLJDD promotes apoptosis and potentially regulates the ferroptosis pathway. QCWZD regulates the crosstalk between the intestinal microbiota and GSDME-mediated pyroptosis.

**FIGURE 2:**
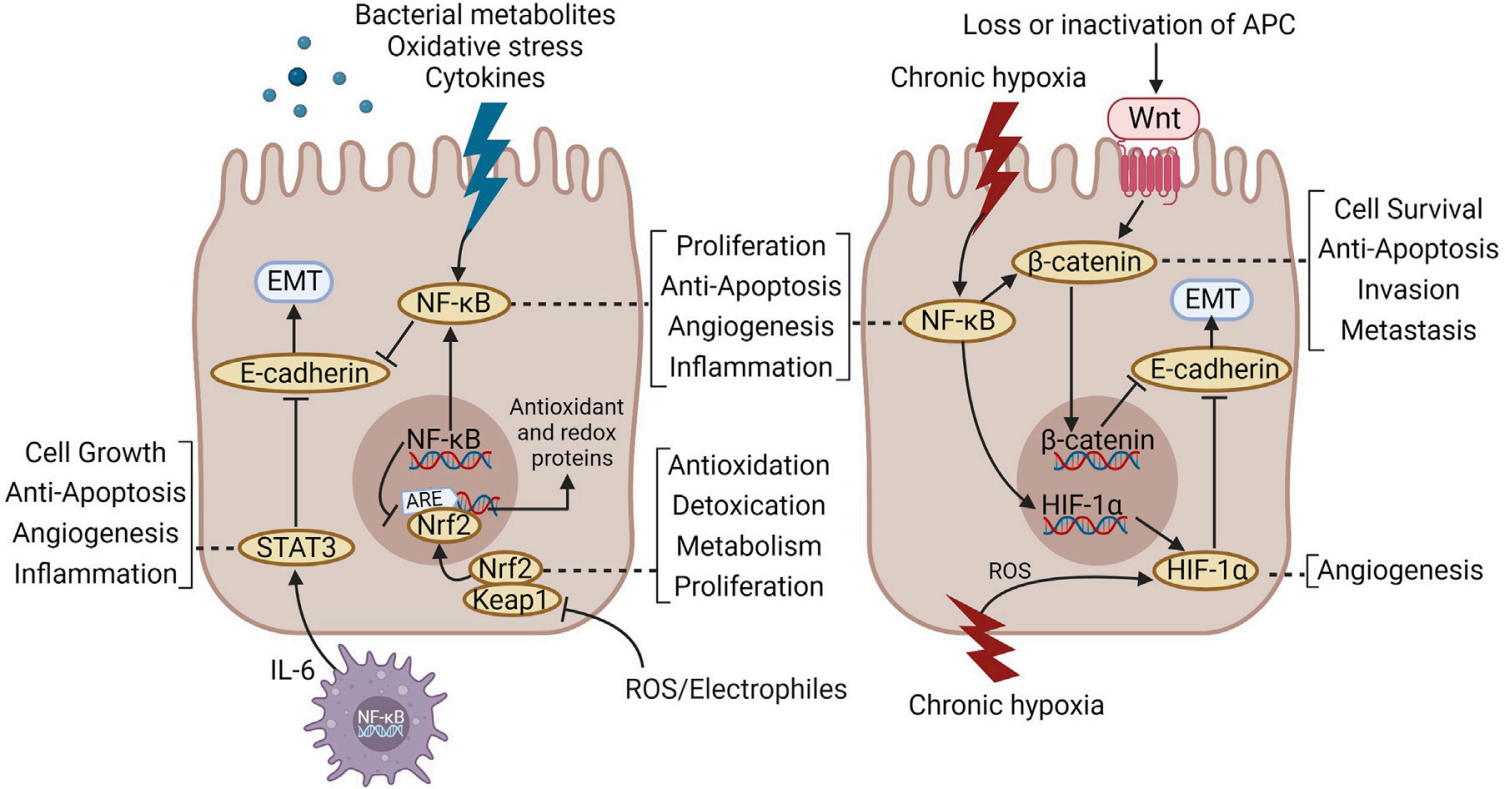
The main signal pathways involved in the treatment of CAC with TCM formulas.

Several classical signal pathways including NF-κB, STAT3, Wnt/β-catenin, HIF-1α, and Nrf2 are majorly involved in the mechanism of TCM formula. Interestingly, NF-κB and its cascades with other signaling pathways have been covered in nearly all studies, thereby forming an NF-κB-centered signaling network (Figure 2). As the core regulator of the inflammatory response, NF-κB is chronically activated in IBD and promotes the development of CAC as a major participant (O'Connor et al., 2010). STAT3 is activated by IL-6 produced by myeloid cells in the TME, with a vital effect on promoting tumor cell proliferation, angiogenesis, and apoptosis inhibition (Yu et al., 2009). Interestingly, IL-6 functions in an NF-κB-dependent manner (Hirano et al., 2020), thereby constituting the NF-κB/IL-6/STAT3 signaling cascade, a “positive feedback loop” (Iliopoulos et al., 2009) that will further expand and preserve the inflammatory signal in CAC to accelerate the progression of IBD-CAC (Liang et al., 2013). At the same time, NF-κB can also activate Wnt/β-catenin and induce the colonic epithelium to acquire the capacity of tumor-initiating cell dedifferentiation, thereby accelerating the occurrence of colorectal cancer (Schwitalla et al., 2013). Moreover, NF-κB and HIF-1α have a close cooperative relationship in tumor hypoxia and the inflammatory microenvironment primarily manifested as mutual crosstalk in various pathways of apoptosis, angiogenesis, and EMT (D'Ignazio et al., 2017). Nrf2 is an important protective factor against oxidative stress. Studies have shown that Nrf2 deficiency promotes DSS-induced colitis (Khor et al., 2006) and AOM/DSS-induced CAC (Li et al., 2008), whereas NF-κB can be inhibited by different Nrf2 activators. This suggests that the crosstalk between the two may have an important effect on the conversion of cellular stress signals into anti-inflammatory responses (Jeong et al., 2004). Therefore, TCM formulas regulate the network comprising multiple signaling pathways with NF-κB as the core, and TCM medicine may play a role in regulating its crosstalk.

We believe that the TCM formula has benefits in regulating TME and maintaining intestinal homeostasis. This can be explained from two aspects. On one hand, TCM formulas can regulate TME outside the cells. On the other hand, TCM formulas can promote PCD in abnormally differentiated cells. Both of these two points help maintain homeostasis in the intestinal environment. The TCM formula can regulate the NF-κB-centered signaling network with features of multiple pathways and targets. Therefore, the TCM formula may be a promising therapeutic drug for regulating colitis-cancer transformation.

However, there are still many shortcomings in the research on the prevention and treatment of CAC with the TCM formula: 1) The design of the experimental protocol was relatively rough, and the research was only limited to the phenomenon level, without targeted verification of the action mechanism. 2) It is challenging to display the therapeutic effect of the TCM formula and the dose-effect connection since many studies have not established the concentration gradient of the intervention drugs and the positive control drugs. 3) The names of Chinese medicinal materials were not standardized enough, and the description of the extraction process was rough, which reduced the repeatability of the experiment. 4) The effect of the TCM formula on the prevention and treatment of CAC has only been verified in basic experiments, and no randomized controlled clinical study with a high level of evidence has been conducted. 5) The intervention model could not simulate the TCM syndrome types and could not reflect the characteristics of TCM syndrome differentiation. 6) The lack of multi-channel validation in the study cannot fully reflect the advantages of multi-channel and multi-target of TCM formula.

In summary, the TCM formula may be a promising treatment candidate for controlling colitis-cancer transformation, however more high-quality research evidence is required to support this claim. Future research needs more scientific and rigorous experimental design to clarify the dose-effect relationship and action target of the TCM formula; Vigorously promote the experimental study of the combination of disease and syndrome in TCM to clarify the mechanism of TCM formula treatment based on the differentiation of the syndrome; With the help of emerging multi-omics technology, a comprehensive exploration of intervention targets of Chinese medicine prescriptions is conducted. Scientifically, objectively, and systematically explore the effects and mechanisms of TCM formulas in the prevention and treatment of CAC in order to provide evidence for further clinical research.
